# The Predictive Effect of Parental Adult Attachment on Parent–Adolescent Attachment: The Mediating Role of Harsh Parenting

**DOI:** 10.3389/fpsyg.2021.710167

**Published:** 2021-08-11

**Authors:** Mengge Li, Xin Chen, Huoliang Gong, Wanghua Ji, Wei Wang, Shifeng Liang, Anran Kong

**Affiliations:** ^1^School of Psychology, Henan University, Kaifeng, China; ^2^School of Management, Henan University of Traditional Chinese Medicine, Zhengzhou, China; ^3^Ruzhou Third Junior High School, Ruzhou, China

**Keywords:** paternal adult attachment, maternal adult attachment, paternal harsh parenting, maternal harsh parenting, father-adolescent attachment, mother-adolescent attachment

## Abstract

This study examines the relationship between parental adult attachment and parent–adolescent attachment and investigates the mediating role of harsh parenting from the perspective of family systems theory. The sample included adolescents and their parents from 1,030 families in Henan Province, China. Measures included the Experiences in Close Relationships Scale, Inventory of Parent and Peer Attachment Scale, and Harsh Parenting Scale. The results showed that paternal avoidance negatively predicts father–adolescent and mother–adolescent attachments, while maternal avoidance and maternal anxiety negatively predict father–adolescent attachment and mother–adolescent attachment, respectively. Paternal harsh parenting not only mediated the relationship between paternal adult and father–adolescent attachment but also between paternal adult and mother–adolescent attachment. However, maternal harsh parenting only plays an intermediary role between maternal adult and mother–adolescent attachment. The results of the study show that compared with the adverse effects of maternal adult attachment and maternal harsh parenting on parent–adolescent attachment, paternal avoidance and paternal harsh parenting more negatively affect parent–adolescent attachment, which is not conducive to familial harmony.

## Introduction

Independence and autonomy gradually develop during adolescence. However, because adolescent psychology remains relatively immature, adolescents exhibit the paradoxical characteristics of wanting autonomy and depending on their parents. This contradiction makes parent–adolescent attachment, that is, the stable, lasting, and deep emotional bond formed between adolescents and their parents (Ainsworth and Bowlby, 1991), complicated and difficult to grasp (Armsden and Greenberg, 1987; Steinberg and Morris, 2001). Notably, the quality of parent–adolescent attachment affects adolescent mental health as well as family harmony (Barcaccia et al., 2020; Wang et al., 2020) and can significantly impact how the adolescent will go on to experience love, marriage, and family in adulthood (Fraley and Roisman, 2019).

Previous studies on adolescent attachment to parents primarily focus on how different forms of attachment impact healthy growth in adolescents. To date, researchers have not sufficiently examined the factors influencing the quality of parent–adolescent attachment. While Hou et al. (2020) explored how the mother's behavior affects her attachment with the autistic child, they did not consider how the father's behavior impacts his own attachment with the child; however, on a related note, Cabrera (2019) observed that a father's interactions with his child meaningfully impact the child's attachment style. Additionally, Jones and Cassidy (2014) explored the relationship between the parent's experiences with a secure base and the adolescent's own experiences of a secure base; however, this study separately explored father–adolescent and mother–adolescent relationships instead of combining them into one model. This composition is problematic because it forgets family systems theory's recognition that father–mother interactions affect parent–adolescent attachment (Weeland et al., 2021), which is discussed in more detail in section Parent Adult Attachment Style and Parent–Adolescent Attachment.

To comprehensively examine the formation mechanism of an adolescent's attachment to their parents, this study investigated the attachment from the family systems theory perspective of family interaction. This study is the first to include the father, mother, and adolescent in one model to investigate the influence of the parents' attachments on the adolescent's attachments to the parents. In examining the relationship between parent adult attachment style and parent–adolescent attachment, this study also looked at whether harsh parenting mediates the impact of the parent's adult attachment style on the parent–adolescent attachment; notably, this study is the first to inquire into the mediating role of harsh parenting.

### Parent Adult Attachment Style and Parent–Adolescent Attachment

Hazan and Shaver (1987) first extended attachment theory to adulthood, suggesting that the emotional connection to a romantic partner can also be regarded as an adult attachment (Hazan and Shaver, 1987). Brennan et al. (1998) proposed the dimensionality of attachment, dividing it into two continuous dimensions: anxious and avoidant. Individuals with an anxious attachment style are afraid of being abandoned and desire close relationships, while those with an avoidant style are more inclined to alienate their partners. Based on the dimensional view, several studies have found that attachment style is an important predictor of personal physical and mental health and the quality of interpersonal relationships (Mikulincer and Shaver, 2008; Liang and Guo, 2014; Molero et al., 2016).

In the field of parenting, the parent's attachment style is an important factor of parenting quality and parent–adolescent attachment (Jones et al., 2014a). Some studies evidence a negative correlation between maternal adult attachment and the adolescent's attachment to their mother (Zhu et al., 2018; Li et al., 2020). Meanwhile, Bi et al. (2018) study of fathers and adolescents found that avoidant and anxious attachment styles in fathers were associated with the quality of the father–adolescent attachment. Another study examining the parent's own adult attachment style and the parent's relationship with the adolescent (from the perspectives of both fathers and mothers) showed that the attachment style the father exhibited toward the mother affected the quality of father's relationship with the adolescent, but not the mother's relationship with the adolescent. Only when fathers, mothers and adolescents are included in the same model can we truly explore the formation path of parent-adolescent attachment. However, because parents were the subject of Lai et al. (2019) study, the study did not offer insights into adolescent attachment to parents. The deeper point here for our purposes is that previous studies have only explored how the parent's attachment style impacts the parent's attachment to the adolescent; no research has yet uncovered how the parent's attachment style impacts the adolescent's attachment to the parent.

According to family systems theory, the family system is composed of different subsystems that influence and interact with each other, such as the marriage, father–child, and mother–child subsystems. Adult attachment belongs to the husband–wife subsystem, which reflects the internal interaction of the family system (Erel and Burman, 1995). A marital relationship largely depends on the attachment quality of both parties, which contributes to the parent–adolescent attachment that reflects the internal interaction of the parent–child subsystem (Belsky, 1984). There are currently hypotheses in family systems theory regarding the influence of the conjugal subsystem on the parent–child subsystem, namely, the spillover and crossover hypotheses. The spillover hypothesis holds that emotions or behaviors generated in one subsystem (e.g., the husband–wife subsystem) are similarly expressed in another (e.g., the parent–child subsystem) (Erel and Burman, 1995). For example, the father's negative association with adult avoidance and anxiety may be reflected in the father–adolescent attachment, and the mother's negative association with adult avoidance and anxiety may be reflected in the mother–adolescent attachment. The crossover hypothesis states that the emotions or behaviors of one of the interacting parties in a certain subsystem of the family (e.g., the feelings of the father in the husband–wife subsystem) will affect those of other parties in other subsystems (e.g., the mother in the mother–child subsystem) (Bolger et al., 1989). For example, the father's negative association with adult avoidance and anxiety may be reflected in the mother–adolescent attachment, and the mother's negative association with adult avoidance and anxiety may be reflected in the father–adolescent attachment.

Drawing on the above evidence, we developed Hypothesis 1: The parents' adult attachment style negatively predicts the parent–adolescent attachment.

### Harsh Parenting as Mediator

Harsh parenting mainly manifests as physical attacks (e.g., slaps, spanking), verbal attacks (e.g., insults and yelling), and other crude behaviors, emotions, and attitudes (e.g., boredom, indifference, anger, emotionality, and insensitivity) toward the children (Wang et al., 2016). Harsh parenting may mediate the effects of parent attachment styles on parent–adolescent attachment. For example, mothers with adult anxiety use over-activation strategies to deal with intimate relationships and may demonstrate more controlling and interfering behaviors toward their adolescent children; notably, such behavioral patterns can easily lead to mother–adolescent relationship conflict (Adam et al., 2004; Selcuk et al., 2010), which has been related to abusive parenting by the mother (Goldberg and Scharf, 2020). Mothers who demonstrate maternal avoidance tend to adopt a deactivation strategy in rearing adolescents, and are more likely to ignore the needs of adolescents and demonstrate an apathetic coping style (Berlin et al., 2011; Mills-Koonce et al., 2011). Mother–adolescent conflict is also likely to increase if the adolescent has unmet needs, and, as noted above, such conflict increases the likelihood that the mother will demonstrate harsh parenting behavior. Previous studies have explored the possible relationship between the mother's adult attachment style and harsh parenting, but no work has been done on the possible link between the father's adult attachment style and harsh parenting.

The spillover hypothesis of family systems theory assumes that both the father and mother are the primary caregivers of an adolescent. Avoidant and anxious adult attachment styles in both parents increase the likelihood that negative emotions and behaviors will characterize the parents' relationships with each other (e.g., the husband–wife subsystem). Parent–parent attachment styles can spill over into the parent–child subsystem, affecting the quality of the interactions between parents and adolescents (Erel and Burman, 1995) and, therefore, the likelihood of harsh parenting as well. For example, if a father has an anxious or avoidant adult attachment style, they may be more likely to demonstrate harsh paternal parenting behaviors. When parents adopt harsh approaches to raising their teenager, the adolescent may feel disrespected and misunderstood—such feelings can cause the teenager to feel dissatisfied with their parents, which weakens the parent–adolescent relationship (Allen and Manning, 2007; Koehn and Kerns, 2017). In step, previous studies have shown that harsh parenting can affect the quality of parent–adolescent attachment (Wang et al., 2020). Helpful to note here is that the crossing hypothesis of family systems theory reasons that one parent's harsh parenting style may not only affect their attachment with the adolescent, but also the other parent's attachment with the adolescent; indeed, Wang et al. (2019) confirmed this hypothesis.

Drawing on the above evidence, we developed Hypothesis 2: Harsh parenting mediates the effect of the parents' adult attachment style on parent–adolescent attachment.

### Current Study

**Figure 1:**
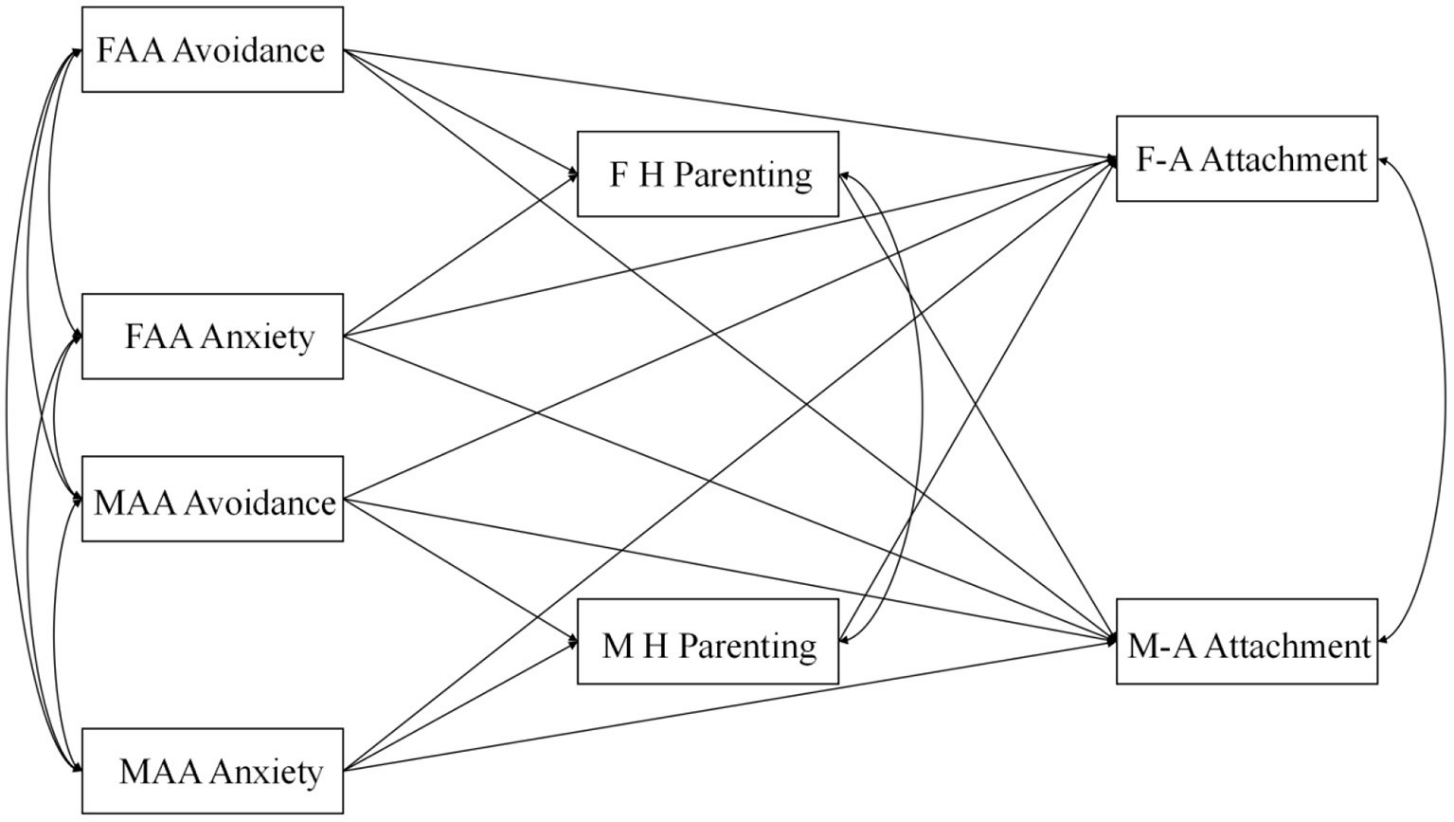
Hypothesis model. FAA Avoidance, father adult attachment avoidance; FAA Anxiety, father adult attachment anxiety; MAA Avoidance, mother adult attachment avoidance; MAA Anxiety, mother adult attachment anxiety; F H parenting, father harsh parenting; M H parenting, mother harsh parenting; F-A Attachment, father-adolescent attachment; M-A Attachment, mother-adolescent attachment.

Ultimately, to respond to the gaps in the research identified above, this study had two key aims. First, it sought to identify the impact of the parents' adult attachment on the adolescent's attachment to the parents in light of family systems theory's crossover hypothesis. Second, the study sought to uncover the relationship between harsh parenting and the adolescent's attachment to the parents. The hypothesis model diagram of this study is shown in Figure 1.

## Method

### Setting and Participants

Participants included parents and adolescents from Henan Province. The sample of families (including adolescents, fathers, and mothers) was created by applying convenience sampling to adolescents from 13 schools in seven different cities in Henan Province, China; namely: Zhengzhou, Kaifeng, Ruzhou, Zhoukou, Lushan, Xingyang, and Shangjie. A total of 1,030 families participated in the survey.

The adolescents in the sample were between 12 and 16 years of age, with an average age of 13.57 (SD = 1.20). Additionally, the adolescent sample comprised 535 boys (51.94%) and 495 girls (48.06%). Most adolescents sampled had siblings (93.60%) and lived with their mothers (86.6%) and fathers (74.10%). Meanwhile, the average ages of the fathers and the mothers in the sample were 42.42 (SD = 5.47) and 40.61 (SD = 5.67).

### Procedure

Data were collected in January 2021 by asking adolescents and their parents to complete questionnaires. Prior to data collection, trained research assistants introduced the study to the adolescents in their classrooms. The legal guardians/close relatives of the participants provided written informed consent. Participants were advised that they were free to withdraw from the study at any time. The questionnaires were administered to the sample only after the consent forms were signed. The adolescents were instructed to independently complete the questionnaires, which were handed out and collected in class. While in school, the adolescents were also given questionnaires to bring home to their parents. Parents were asked to first provide demographic information about the family and themselves and then to complete the materials (described in section Measures) that measured adult attachment and harsh parenting. After the surveys were completed, the child brought the questionnaires back to school. Incomplete questionnaires were excluded; ultimately, 80.7% of the original participants completed the questionnaires.

The study was approved by the Research Ethics Committee of the Institute of Psychology and Behavior at the author's university and conducted with the permission of the principals of the participating schools.

### Measures

#### Parent-Reported Adult Attachment

The Experiences in Close Relationship Scale (ECR), revised by Li and Kato (2006) (ECR-R), was used to examine adult attachment. The ECR-R is a 36-item self-report questionnaire that measures attachment anxiety (e.g., “I worry about being abandoned”) and attachment avoidance (e.g., “I prefer not to show a partner how I feel deep down”). The participants responded to the items on a 7-point Likert scale ranging from 1 (strongly disagree) to 7 (strongly agree); higher scores indicate higher attachment anxiety or avoidance. In this study, the Cronbach's α coefficients of the paternal attachment avoidance and attachment anxiety scales were 0.80 and 0.90, respectively. The Cronbach's α coefficients of maternal attachment avoidance and attachment anxiety scales were 0.81 and 0.91, respectively.

#### Parent-Reported Harsh Parenting

The Harsh Parenting Scale, revised by Wang (2017), was used to examine harsh parenting. It is a 4-item scale (e.g., “When this child does something wrong or makes me angry, I “lose my temper or even yell at them”). The participants responded to the items on a 5-point Likert scale ranging from 1 (strongly disagree) to 5 (strongly agree); higher scores indicate harsher parenting. The reported Cronbach's alphas for fathers and mothers were 0.91 and 0.90, respectively.

#### Adolescent-Reported Parent–Adolescent Attachment

We adopted items on father–and mother–adolescent attachment selected from the Inventory of Parent and Peer Attachment, which was developed by Armsden and Greenberg (1987) and revised by Li et al. (2009). It has 13 items across three dimensions: trust, communication, and alienation (e.g., “My father/mother can tell when I am upset about something.”). The participants responded to the items on a 5-point Likert scale ranging from 1 (strongly disagree) to 5 (strongly agree). Higher scores for the adolescent's attachment to their parents indicate a better relationship between the parents. The reported Cronbach's alphas for the father–adolescent and mother–adolescent attachment scales for adolescents were 0.89 and 0.85, respectively.

#### Family Socioeconomic Status

We asked parents to report their occupations and education levels as well as the family's monthly income. As in prior studies (Shi and Shen, 2007), parental educational attainment was coded from 1 (primary school and below) to 5(graduate education or above). Regarding educational attainment among the fathers, 78.5% were middle school graduates, 9.5% were high school graduates, and 12.0% held college degrees or above. Meanwhile, among the mothers, 73.9% were middle school graduates, 9.5% were high school graduates, and 16.6% held college degrees or above. Parental occupations was coded from 1 (temporary workers, unemployed, unskilled and agricultural working classes, such as farmers) to 5 (professional senior managers and senior professional and technical personnel members and professional directors, including those in the Party and government, institutions and social organizations leading cadres in government organs, such as civil servants, company managers, foremen, etc). Participants were asked to report their monthly income by selecting from 1 (below 1000) to 7 (above 10000). Annual household income was calculated by multiplying monthly household income by 12. The mean annual household income in the sample was 40328.16 yuan (SD = 27065.86). Finally, the parents' occupational levels and education levels and the family income levels were added together to yield a total score used to indicate family socioeconomic status (Family SES). The mean Family SES was 10.28 (SD = 3.92).

### Data Analysis

Data were analyzed using IBM SPSS version 22 and Mplus 7.4. The analyses were conducted in two steps. First, descriptive statistics (i.e., M and SD) and bivariate correlations for the major variables were calculated. Second, a bootstrapping method with the Mplus 7.4 was used to evaluate the mediation model. The 95% confidence interval (CI) for the indirect effect was a bias-corrected estimate based on 5,000 bootstrapping resamples. The mediating effect was considered significant at the level of *p* < 0.05 when the 95% CI did not include zero. Missing data were <1% and estimated using expectation maximization.

## Results

### Descriptive Statistics and Correlations

Table 1 presents the means and standard deviations of all study variables and the results of the correlation analyses. The correlation matrix indicated that adolescent age was positively related to maternal adult anxiety. Meanwhile, adolescent age was negatively related to paternal adult anxiety and paternal harsh parenting. Family SES was not related to other variables. Both paternal and maternal avoidance and anxiety were positively and significantly related to harsh parenting, that is, the higher the father's and mother's avoidance and anxiety, the harsher their parenting styles. Both father's and mothers' levels of avoidance and anxiety were negatively and significantly related to parent–adolescent attachments, indicating that the higher the levels, the worse the parent–adolescent attachment development. Harsh parenting by both fathers and mothers was negatively and significantly correlated with parent–adolescent attachments, indicating that the harsher the parenting style, the worse the parent–adolescent attachment development.

**Table 1:** Descriptive statistics and correlations of the main study variables (*N* = 1,030).

	**1**	**2**	**3**	**4**	**5**	**6**	**7**	**8**	**9**	**10**	**11**
1.Age	1										
2.Gender	−0.04	1									
3.Family SES	−0.16^**^	0.00	1								
4.FAA1	0.01	−0.03	−0.03	1							
5.FAA2	0.00	−0.09^**^	−0.09	0.42^**^	1						
6.MAA1	−0.01	−0.02	0.02	0.63^**^	0.37^**^	1					
7.MAA2	0.07^*^	−0.04	−0.01	0.36^**^	0.58^**^	0.44^**^	1				
8.FHP	0.02	−0.11^**^	0.02	0.35^**^	0.42^**^	0.30^**^	0.30^**^	1			
9.MHP	0.04	−0.05	−0.01	0.31^**^	0.32^**^	0.33^**^	0.44^**^	0.52^**^	1		
10.FCA	0.01	0.05	−0.03	−0.23^**^	−0.12^**^	−0.25^**^	−0.14^**^	−0.22^**^	−0.19^**^	1	
11.MCA	−0.01	0.03	−0.02	−0.26^**^	−0.16^**^	−0.22^**^	−0.22^**^	−0.23^**^	−0.26^**^	0.76^**^	1
M	-	-	-	3.17	3.40	3.20	3.32	2.01	2.15	3.84	3.90
SD	-	-	-	0.88	1.16	0.90	1.17	0.94	0.94	0.72	0.67

*Family SES, Family socioeconomic status; FAA1, Paternal adult attachment avoidance; FAA2, Paternal adult attachment anxiety; MAA1, maternal adult attachment avoidance; MAA2, maternal adult attachment anxiety; PHF, paternal harsh parenting; MHP, maternal harsh parenting; FCA, father-adolescent attachment; MCA, mother-adolescent attachment*.

* *P < 0.05*.

** *P < 0.01*.

### The Direct Effects of Parental Adult Attachment Style on Parent–Adolescent Attachment

Associations between the dependent variable and the independent variables were examined using linear regressions. Structural equation modeling (SEM) was used to evaluate the regression model for parent adult attachment and parent–adolescent attachment using Mplus 7.4. Adolescent age, gender, and family SES were included as covariates in the model. The model fit index was as follows: CFI = 1, χ2 = 0, df = 0.

**Figure 2:**
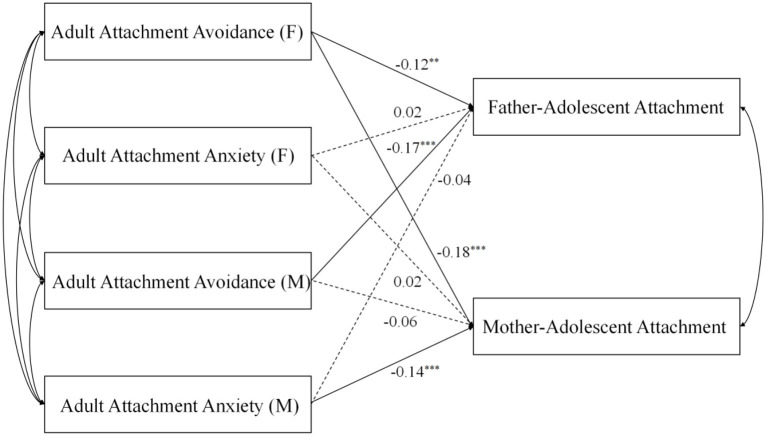
Regression model. F, fathers; M, mothers. **P < 0.01. ***P < 0.001.

As shown in Figure 2, the direct paths from adult anxiety to father–adolescent attachment were not significant for either fathers or mothers (β_father_ = 0.02, β_mother_ = −0.04, *p* > 0.05). The path from paternal adult anxiety to mother–adolescent attachment was not significant (β = 0.02, *p* > 0.05). Meanwhile, the path from maternal avoidance to mother–adolescent attachment was also not significant (β = −0.06, *p* > 0.05). Significant effects of paternal avoidance on parental–adolescent attachment were found; father–adolescent attachment and mother–adolescent attachment were significantly and negatively predicted (β = −0.12, *p* < 0.01, β = −0.18, *p* < 0.001). Maternal avoidance significantly and negatively predicted father–adolescent attachment (β = −0.17, *p* < 0.001), and mother adult anxiety significantly and negatively predicted mother–adolescent attachment (β = −0.14, *p* < 0.001). These results indicated that higher parental avoidance and anxiety were linked to lower levels of parent–adolescent attachment.

### The Mediation Effects of Harsh Parenting

**Figure 3:**
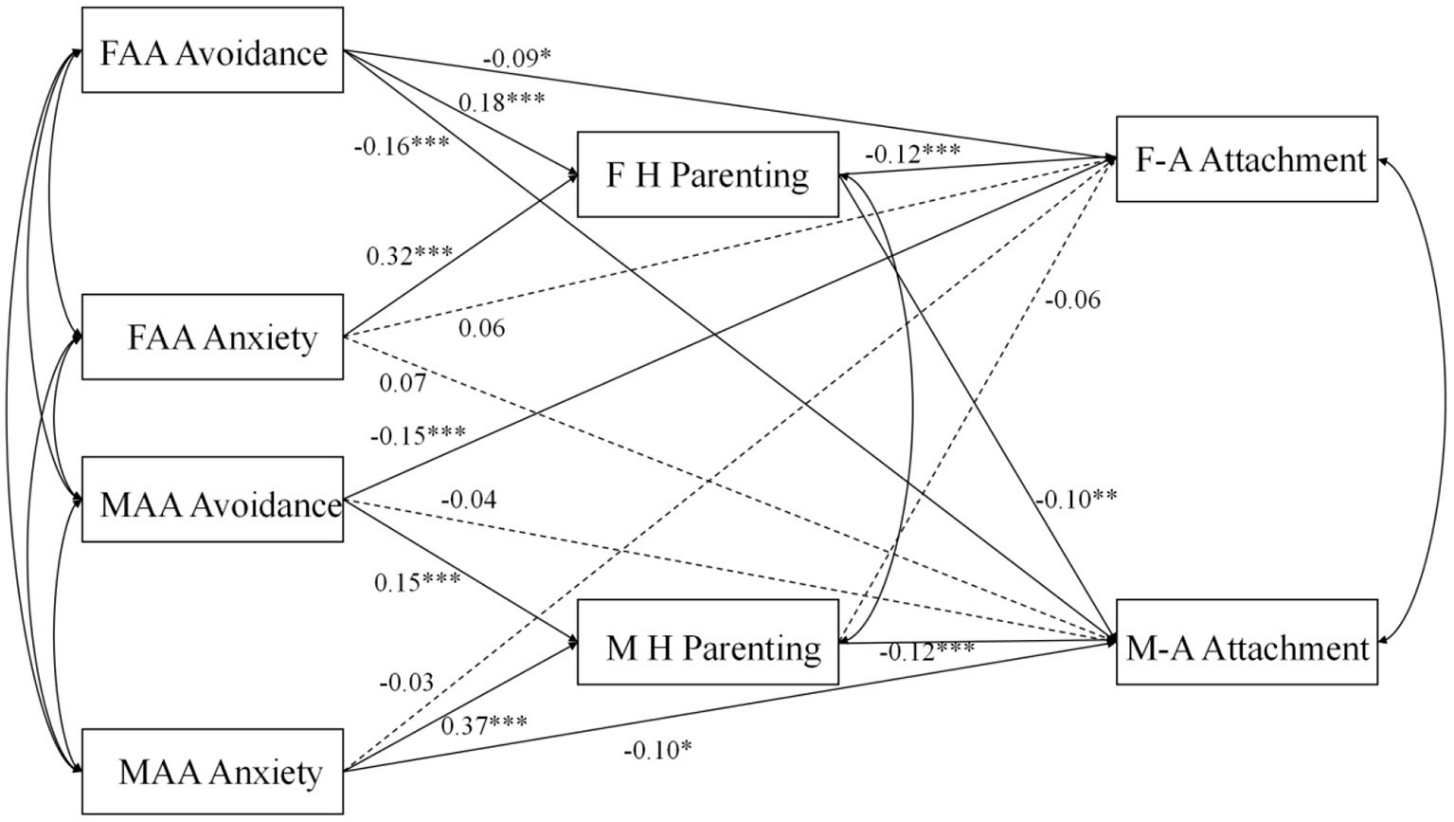
Structural equation model. FAA Avoidance, father adult attachment avoidance; FAA Anxiety, father adult attachment anxiety; MAA Avoidance, mother adult attachment avoidance; MAA Anxiety, mother adult attachment anxiety; F H parenting, father harsh parenting; M H parenting, mother harsh parenting; F-A Attachment, father-adolescent attachment; M-A Attachment, mother-adolescent attachment. *P < 0.05. **P < 0.01. ***P < 0.001.

We used SEM to assess the model of the mediation effects of harsh parenting, using Mplus 7.4. Adolescents' ages, gender and family SES were included as covariates in the model. The model fit index of the research models demonstrated adequate goodness-of-fit statistics: [$\chi_{\left(4\right)}^{2}$ = 6.944, CFI = 0.986, RMSEA = 0.027]. As shown in Figure 3, the indirect impact of parental avoidance and anxiety on parent–adolescent attachment was also indicated by a bias-corrected bootstrap at the 95% CI. The results indicated that paternal avoidance and anxiety were indirectly related to father–adolescent attachment through paternal harsh parenting (95% CI avoidance: −0.04, −0.01; 95% CI anxiety: −0.07, −0.02). Mother avoidance and anxiety were related to father–adolescent attachment through maternal harsh parenting (95% CI avoidance: −0.03, 0.01; 95% CI: −0.06, 0.01). Mother avoidance and anxiety were only indirectly related to mother–adolescent attachment through maternal harsh parenting (95% CI avoidance: −0.04, −0.01; 95% CI: −0.08, −0.02). Father avoidance and anxiety were only indirectly related to mother–adolescent attachment through paternal harsh parenting (95% CI avoidance: −0.04, −0.01; 95% CI: −0.07, −0.01).

## Discussion

### Analysis

The present study aimed to investigate the relationship between parental adult and parent–adolescent attachment and mediate the role of harsh parenting based on the family systems theory. The results largely support the hypothesis of this study that paternal avoidance directly and negatively predicts father–adolescent attachment. These findings are consistent with a previous study on the topic (Jones et al., 2014a), thus enforcing the support for the spillover hypothesis. When interacting with the mother, the father increases avoidance coping styles with paternal avoidance. This approach alienates the relationship between spouses, which is likely to cause dissatisfaction in the wife and create tension in the relationship. The consequent undesirable moods will spill over to the parents' interactions with their teenagers and, in turn, affect parent–adolescent attachment (Erel and Burman, 1995). In Chinese families, fathers spend less time interacting with adolescents than mothers. Avoidant fathers are likely to interact with adolescents by avoiding their emotional intimacy needs, inevitably affecting adolescents' doubts about whether their fathers care and support them. It is difficult for adolescents to obtain positive and effective support from avoidant fathers, which deprives them of a high-quality safety foundation and safe haven, which is the basis for the formation of parent–adolescent attachment (Groh et al., 2016).

Second, there is a significant negative correlation between paternal avoidance and mother–adolescent attachment, which supports the crossover hypothesis that paternal avoidance not only affects their own attachment with the adolescent but also the mother–adolescent attachment. Avoidant fathers show a lack of relative intimacy and trust toward their partners (Rodriguez et al., 2020). This makes wives feel unloved and uncared for in the relationship, more so since women have a greater desire for intimacy and confide in their partners more than men. Moreover, women's marital relationships affect their own sense of value (Siegel et al., 2018). Avoidant fathers are unable to meet their wives' needs for intimacy and value in the marital relationship. Wives are likely to project their unmet needs into the process of having a good relationship with adolescents. At the same time, mothers may seek more intimacy from their adolescents to compensate for its lack in their marriage. Teenagers may want to avoid interactions with their mothers that are full of emotional control and excessive intimacy, which is also not conducive to the development of healthy mother–adolescent attachment.

Third, the present study indicated that maternal adult anxiety significantly negatively predicts mother–adolescent attachment, which is in accordance with previous results (Ceglian and Gardner, 2001; Berlin et al., 2011; Jones et al., 2014b). Maternal adult anxiety may cause the mother to overreact in dealing with teenagers, which makes mothers more controlling in the process of interaction and emotional instability. Mothers with adult anxiety are more likely to develop an internal working model that others perceive negatively, which makes the mother frequently question her self-worth. This can lead to an oversensitivity to signals of threatening relationships, and an overreaction to maintain relationships (Godbout et al., 2019). However, adolescence is a period of enhanced self-awareness, and such a relationship pattern will encourage adolescents to constantly want to escape from their excessively controlling mothers, which will lead to the adverse development of mother–adolescent attachment.

We also learned that harsh parenting has a significant mediating effect between parental adult and parent–adolescent attachment, which is consistent with Hypothesis 2 of this study. These findings are consistent with a previous study (Jones et al., 2014a) and the theory that adolescence is a period in which independent consciousness increases (Steinberg, 2007). It is helpful to recall here that, as noted above, avoidant parents tend to adopt a deactivation strategy when raising teenagers and can be insensitive and unable to appropriately respond to their teenager's needs, which can make the teenager dissatisfied with their parents—a trend that is likely to lead to harsh parenting (Berlin et al., 2011; Mills-Koonce et al., 2011). Meanwhile, anxious parents are likely to overreact to their teenager's needs and, thus, interfere with the normal needs of teenagers; similarly, this can easily lead to dissatisfaction between parents and teenagers and increase the possibility of harsh parenting (Adam et al., 2004; Selcuk et al., 2010). Harsh parenting pollutes benign interactions between teenagers and their parents by normalizing a lack of warmth and support, destroying any safe foundations in the relationship, and eroding the quality of the attachment (Wang et al., 2019, 2020; Havighurst et al., 2020).

The results also show that a father's harsh parenting style mediates his own adult attachments and his attachment with his adolescent as well as the mother–adolescent attachment; however, a mother's harsh parenting only mediates her own adult attachments and her attachment with her adolescent; notably, this suggests that paternal and maternal parenting styles differently impact interactions in a family with an adolescent (Sabey and Rauer, 2017; Siegel et al., 2018; Rodriguez et al., 2020). However, this finding is inconsistent with the results of some existing study (Jones et al., 2014a). Moreover, we found that paternal parenting behavior heavily impacts maternal parenting behavior: a father's negative approach to rearing an adolescent will affect the mother's emotions and, relatedly, the quality of her interactions with and attachment to the adolescent (Newland et al., 2013). In sum, a mother's harsh parenting style does not directly affect father–adolescent interactions and, thus, does not affect father–adolescent attachment; in contrast, a father's harsh parenting style significantly impacts the adolescent's attachments to both parents and family harmony. These findings may speak to Chinese cultural norms: the proverbial father is the head of the household and, thus, is more likely to influence the mother than vice versa; accordingly, mothers may have historically been less likely to have the power to control the parenting style with which their adolescents were raised.

### Limitations and Future Directions

This section acknowledges several limitations of the present study and suggests directions for future research. First, no conclusion regarding causality or directionality can be drawn because of the study's cross-sectional design. Reciprocal relationships between the parents' adult attachment styles and harsh parenting have been proposed; therefore, a longitudinal design may provide more comprehensive insight into the directionality of parent adult attachment, harsh parenting, and parent–adolescent attachment. Second, the research objects in this study were samples from Henan Province in China. This setting's households have relatively low economic statuses compared with households in other developed cities. Therefore, the results of this study cannot be blindly generalized across different contexts. Future studies would do well to include more regions to improve external validity. Furthermore, as time passes and society changes, family structures and activities also change; this study collected data in January 2021, and thus, it will be important for future scholars to interpret the findings according to the time context and conduct studies that update the results in step with societal changes. Finally, our use of a self-reported questionnaire did not preclude the influence of social approval on our subjects. In the future, observation interviews and experiments could be considered to obtain more objective and richer measurements of parent–adolescent attachment.

### Research Implications

This study has implications for research on and practical interventions in parent–adolescent relationships in China and other countries. Theoretically, this study is the first to apply family systems theory to develop a model that includes the father, mother, and teenager to investigate the formation mechanism of parent–adolescent attachment. In step, the study's findings that parent adult attachments can influence adolescent attachments to parents; that paternal and maternal avoidance and anxiety can negatively influence parent–adolescent attachment; that paternal adult attachment can impact mother–adolescent attachment; and that paternal avoidance more significantly impacts family harmony than maternal avoidance lay the ground for future studies on how the adult attachment styles of each parent can affect parenting behavior and parent–adolescent attachment.

Practically, the study's according findings confirm that psychological counselors should recognize the important influence of the parents' adult attachments on the adolescent's attachments to their parents rather than only evaluating relationship difficulties by considering parent–adolescent interactions. Put differently, parent–adolescent attachment issues may be primarily rooted in the parents' own relationships with each other, rather than in the parent's relationship with the adolescent; applying this insight to counseling practice may significantly change best practices for healthy interventions. Along these lines, our finding that paternal adult attachment is often a more powerful determinant of family relationships than maternal adult attachment may enhance family therapy interventions; more specifically, family therapists may find that intervening in paternal avoidance proves more effective than only intervening in maternal attachment styles. Finally, family therapists may also do well to help avoidant and anxious parents improve their ability to regulate their emotions Parents' good mood can reduce the frequency of conflict with teenagers. Helpful interventions may include programs that teach mindfulness to reduce harsh parenting (Mah et al., 2020) and train parents in behaviors that promote healthy parent–adolescent attachment.

## Conclusion

This study found that paternal and maternal adult attachment styles differently impact an adolescent's attachments to their parents and, moreover, that harsh parenting mediates the effect of parental adult attachment on parent–adolescent attachment. Additionally, the results showed that paternal avoidance plays a particularly important role in regulating attachment styles throughout the family. Future family therapy practices in China should focus on intervening in paternal avoidance to nurture the healthy development of parent–adolescent relationships and family harmony.

## Data Availability Statement

The raw data supporting the conclusions of this article will be made available by the authors, without undue reservation.

## Ethics Statement

The studies involving human participants were reviewed and approved by The Research Ethics Committee of the Institute of Psychology and Behavior, Henan University. Written informed consent to participate in this study was provided by the participants' legal guardian/next of kin.

## Author Contributions

ML conceived the research. XC, HG, WW, and WJ designed the research. WW, AK, and SL performed the research and analyzed the data. ML, XC, HG, WW, and WJ contributed to the writing of the manuscript. All authors contributed to the article and approved the submitted version.

## Conflict of Interest

The authors declare that the research was conducted in the absence of any commercial or financial relationships that could be construed as a potential conflict of interest.

## Publisher's Note

All claims expressed in this article are solely those of the authors and do not necessarily represent those of their affiliated organizations, or those of the publisher, the editors and the reviewers. Any product that may be evaluated in this article, or claim that may be made by its manufacturer, is not guaranteed or endorsed by the publisher.
